# Comparison of soil analytical methods for estimating wheat potassium fertilizer requirements in response to contrasting plant K demand in the glasshouse

**DOI:** 10.1038/s41598-017-11681-4

**Published:** 2017-09-12

**Authors:** Yulin Zhang, Gunasekhar Nachimuthu, Sean Mason, Michael J. McLaughlin, Ann McNeill, Michael J. Bell

**Affiliations:** 1 College of Resources and Environment, Northwest A&F University, Yangling, 712100 China; 2 School of Agriculture, Food and Wine, University of Adelaide and the Waite Research Institute, 5064 Glen Osmond, SA Australia; 3 NSW Department of Primary Industries, Australian Cotton Research Institute, Locked Bag 1000, Narrabri, NSW 2390 Australia; 4 CSIRO Sustainable Agriculture Flagship, CSIRO Land and Water, PMB 2, Glen Osmond, SA 5064 Australia; 5 The School of Agriculture and Food Sciences, The University of Queensland, Gatton, QLD 4343 Australia; 6 Queensland Alliance for Agriculture and Food Innovation, St Lucia, QLD 4072 Australia

## Abstract

The traditional soil potassium (K) testing methods fail to accurately predict K requirement by plants. The Diffusive Gradients in Thin-films (DGT) method is promising, but the relationship between the DGT-measured K pool and plant available K is not clear. Wheat (*Triticum aestivum L*., cv. Frame) was grown in 9 Australian broad acre agricultural soils in a glasshouse trial until the end of tillering growth stage (GS30) with different plant K demands generated by varying plant numbers and pot sizes. Different K concentrations in soils were varied by 4 rates of K fertilizer application. The relative dry matter and K uptake were plotted against the soil K test value (CaCl_2_, Colwell and NH_4_OAc and DGT K measurements). To obtain 90% of maximum relative dry matter at low root density (closest to field conditions), the critical value of the NH_4_OAc K method was 91 (R^2^ = 0.56) mg kg^−1^. The DGT K method was not able to accurately predict relative dry matter or K uptake due to a weak extraction force for K from soils with high CEC values. Further endeavor on increasing K extraction force of the DGT method is warranted to obtain accurate plant available K results.

## Introduction

Accurate soil testing methods for measuring “plant-available” K would not only maximize crop productivity and quality, but also avoid long term K depletion of fertile farm lands such as what has occurred in grain cropping regions of western and eastern Australia^1^. To achieve these aims, scientists have long been studying K forms in soils and the mechanisms of K uptake by plant roots. The different (operationally defined) forms of K in soils are solution K, exchangeable K, non-exchangeable K (slowly exchangeable K) and mineral K, with K present in these pools in equilibrium with each other^2, 3^. Plant available K refers to mainly soluble K and exchangeable K^4^, although some non-exchangeable K and mineral K can become soluble or exchangeable during a plant growing season. Plants take up K mainly from soil via roots, as K foliar application is not widely employed. Barber *et al*. proposed that K reached plant roots mainly by diffusion^5^, and subsequent work by Barber showed that K taken up by corn roots supplied by diffusion accounted for 80% of the overall total K uptake in a fertile Alfisol silt loam^2^. Other evidence, provided by Mills *et al*., suggested that approximately 85% of the K moved to root surfaces by diffusion through water films around soil particles^6^. Further, Baligar reported that diffusion contributed more to K uptake by plants than mass flow, with 99% and 96% of total K uptake occurring by diffusion for corn (20 days growth) and wheat (25 days growth), respectively^7^. Therefore, it is apparent that plants take up K mainly from the soluble and exchangeable K pools from soils with most of the plant available K moving from the soil solid phase to root surfaces by diffusion. As most K is taken up by roots, the size and shape of roots affect K uptake to a large extent. It was reported that potassium-deficient plants had reduced root to shoot dry weight ratios^8, 9^. Fusseder and Krauss reported that K uptake by maize in field conditions decreased from 50% to 12% of the possible theoretical uptake based on a mathematic treatment of data when root density varied from >2 cm cm^−3^ to <2 cm cm^−3^ ^10^. Consequently, it becomes difficult to predict plant K requirements accurately using soil testing alone as the root growth of plants is unpredictable and dependent on many other factors, such as soil bulk density, soil compaction, soil moisture, temperature and light, other nutrient supply, microorganism activity, etc.

The most common soil tests for predicting available soil K are soil solution extraction and various chemical methods that attempt to either just displace K from the cation exchange complex (exchangeable K), or in addition, measure some of the non-exchangeable K that may contribute to plant uptake during the season (extractable K). The latter tests generally require stronger extracts, and except for extraction with 0.5 mol L^−1^ NaHCO_3_ (the Colwell K soil test, reported by Rayment and Lyons to be the most widely used method for measuring available K in Australia^11^), are only used in specific industries (e.g. sugarcane) or regions (Victoria) where calibrations have been developed^12^. The solution K method measures K in soil solution and easily exchangeable K from the soil solid phase using extraction in water or 0.01 mol L^−1^ CaCl_2._ There are a variety of chemical extraction methods that attempt to measure exchangeable K, but in Australia these typically rely on rapidly replacing K^+^ on the exchange sites using NH_4_^+^, Ba^2+^ or Na^+^^12^. An ion-exchange resin method was used for available K measurement in soils^13^, and the resin K was linearly correlated with the NH_4_OAc K results (R = 0.97), illustrating that they measure a similar K pool in soils. A resin disc method allowed direct contact of the resin disc and soil paste, but a poor regression between K uptake and the quantity of K adsorbed by the resin was observed due to luxury K supply associated with the process of direct contact of the disc and soil sample^14^. Potassium supply rate was measurable using a plant root simulator (PRS, a cation exchange membrane method), expressed as weight per contact area per burial period. However, the sensitivity of the membrane to changes in soil K suppy over time was reported to be the obstacle for simple interpretation of the supply rate measurements^15, 16^.

It is well established that soil solution K measured using the CaCl_2_ K method represents an intensity factor while exchangeable K measured using extractants like NH_4_OAc represents a quantity factor^4, 17^. Barber reported that K uptake is influenced more by the K quantity factor at high root density situations while K uptake is influenced more by the K intensity factor at low root density situations^18^. Bell *et al*. reported that soil solution K values varied by 6–7 fold in different soil types despite similar exchangeable K contents^19^, so it can be concluded that measures of K intensity and quantity differ greatly across soils with varying soil properties. In particular, soil clay content influence the K levels observed in different extraction methods. For example, Houba *et al*. acknowledged that CaCl_2_ did not extract all of the exchangeable K from soils, especially soils with high clay contents^20^. In a glasshouse trial using Guinea grass, Darunsontaya *et al*. suggested that the NH_4_OAc-K method predicted K availability more accurately than other extraction methods (water, HNO_3_, HNO_3_-NH_4_OAc) and the total K method^21^. However, Krishna reported that response of wheat to K application predicted using the exchangeable K method was soil type dependent, and more specifically related to the K buffering ability associated with clay content^22^. Similarly, Gourley *et al*. reported that even commonly used extractable K measures like the Colwell K method were also soil type dependent, with pasture response to K application showing the critical Colwell K values increased with clay content^23^.

The Diffusive Gradients in Thin-films (DGT) technique has been successfully used to more accurately predict plant available phosphorus (P) compared to traditional extraction methods^24–28^. The theory of element uptake by the DGT from soil samples has been extensively discussed^29–33^. Simply, when a DGT device is deployed on water-saturated soil samples, the target element in soil solution diffuses through the diffusive gel and accumulates in the binding gel. When the element concentration at the DGT surface is lowered by uptake by the binding gel, the element from the soil solid phase desorbs to replenish this depletion. Therefore, the fraction of an element measured by the DGT is assumed to incorporate the soluble pool and part of the insoluble pool from the soil solid phase. Tandy *et al*. proposed that the DGT method could be used for soil K measurement by using Amberlite IRP-69 resin as the binding gel and found a similar accuracy to the NH_4_OAc K method to predict K concentrations in winter barley grown in pots^34^. Zhang *et al*. optimized the resin gel by using a mixed Amberlite and ferrihydrite (MAF) gel (to allow simultaneous measurement of P and K) and the MAF gel showed advantages over the resin gel used by Tandy *et al*.^35^. More extensive quantification of the effects of competing cations on K uptake by the DGT will facilitate assessment of the accuracy of the DGT method in predicting plant growth response to K application.

The aims of this research were to 1) investigate the accuracy of DGT K relative to existing soil K tests for predicting wheat response (growth and plant K uptake) to fertilizer K application in different soils types with a range of plant available K concentrations; and 2) compare the predictive performances of these soil tests under differing plant K demands and root densities.

## Methods

### Soil characterization

Nine soils from typical grain producing areas across Australia with known low available K concentrations were dried and sieved to ≤2 mm for use in a glasshouse trial. The soils varied in texture and other inherent soil properties in an attempt to capture soil types prone to K deficiency in Australia (Table 1). Soil pH was measured using 0.01 mol L^−1^ CaCl_2_ solution with a soil to solution ratio of 1:5^36^; organic carbon was measured using the Walkley and Black method^36^; exchangeable cations (Ca, Mg, Na and K) and cation exchange capacity (CEC) were extracted using 1 mol L^−1^ NH_4_OAc^36^; soil particle size was determined using the method described by Bowman and Hutka^37^; and water holding capacity was measured using the same method as Jenkinson and Powlson^38^.

**Table 1:** Basic physical and chemical properties of the soils, and soil K testing values on control soils using different soil testing methods.

Site	Abbreviation	State	EC (µS cm^−1^)	pH	Organic Carbon (%)	CaCl_2_ K (mg kg^−1^)	Colwell K (mg kg^−1^)	NH_4_OAc K (mg kg^−1^)	DGT K (mg L^−1^)	Exchangeable Ca (cmol kg^−1^)	Exchangeable Mg (cmol kg^−1^)	CEC (cmol kg^−1^)	Clay (%)	Sand (%)	Silt (%)
Karoonda	KD	SA	93	6.43	0.28	73	71	77	7.2	0.7	0.2	1.0	3	95	2
Lake Bolac	LB	VIC	90	5.78	1.33	71	69	76	10.0	1.9	0.3	2.4	3	92	4
Ngarkat	NK	SA	17	6.55	0.62	20	21	22	2.6	0.9	0.1	1.0	4	94	2
Gindie B	QB	QLD	47	6.95	0.47	11	28	34	0.6	9.6	7.1	17.1	67	18	15
Capella B	QC	QLD	48	7.04	0.60	21	81	94	1.0	18.0	8.9	17.3	68	20	12
Kingaroy	QL	QLD	59	5.30	1.11	34	65	56	1.9	2.3	1.3	27.5	41	43	16
Jandowae	QS	QLD	67	6.01	0.51	44	100	135	1.2	7.2	6.2	14.9	66	12	22
Regans Ford	RF	WA	141	6.14	2.17	48	52	56	6.4	3.1	0.3	3.6	4	94	2
Wickepin	WN	WA	69	5.32	0.81	32	47	37	3.9	0.7	0.1	1.0	6	88	6

### Glasshouse trial

A glasshouse trial was designed to investigate the effects of differing plant demand and root density on K uptake by wheat, established by growing plants in two contrasting soil volumes with differing plant densities, and to assess whether these parameters affected the wheat response to K fertilizer and to compare the soil analytical methods to estimate wheat K fertiliser requirements. The soils were amended with nutrient solutions containing 100 mg kg^−1^ of nitrogen (as NH_4_NO_3_), 3 mg kg^−1^ of copper (as CuSO_4_•5H_2_O), 5 mg kg^−1^ of magnesium (as MgSO_4_•7H_2_O), 3 mg kg^−1^ of manganese (as MnSO_4_•H_2_O) and 10 mg kg^−1^ of zinc (as ZnSO_4_•7H_2_O), which altogether resulted in a total of 15 mg kg^−1^ sulfur. Phosphorus (as H_3_PO_4_) was applied at different rates for each soil to provide sufficient available P for wheat plants based on phosphorus buffering index (PBI) values and initial P status as assessed by DGT P^25^. In order to obtain a plant dry matter response curve for wheat, K (as KCl) was applied at 4 different rates (Table 2) in each soil type. After addition of nutrient solutions, the soils were thoroughly mixed and incubated for 2 days. The pots were sealed with plastic bags to prevent moisture loss and nutrient leaching. Initially 500 g of each soil (3 replicates) was placed into a small pot (12 cm diameter at the top, 10 cm diameter at the bottom, 11 cm high), another 1250 g of each soil (3 replicates) was placed into a large pot (14 cm diameter at the top, 11.5 cm diameter at the bottom, 14.5 cm high) and 100 g was left as a subsample for soil analysis.

**Table 2:** Root densities and wheat dry matter in response to K application; SD means the standard deviation; DM means dry matter; RDM means relative dry matter and R^2^ is the coefficient obtained by fitting the Mitscherlich curve, where “—” means no R^2^ obtained due to: a) the response in the fertilized treatment was unexpectedly below the controls, dry mass in the control soil is taken as the DM_max_ and b) a linear response was observed, dry mass of 120% of the highest K rate is taken as the DM_max_; different letters mean significant difference observed at P ≤ 0.05.

Soil	Pot size	K rate (mg kg^−1^)				P (mg kg^−1^)	Root density (g m^−3^)	SD of root density	DM_control_ (g pot^−1^)	RDM of control soils (%)	DM_max_ (g pot^−1^)	DM_max_ predicted (g pot^−1^)	R^2^
		Control	K 1	K 2	K 3								
KD	Small	0	50	150	300	300	169bcd	74	0.39bcd	75	0.59	0.53	0.64
LB		0	50	150	300	300	102abc	20	0.34abc	83	0.41	0.41	0.73
NK		0	50	150	300	300	49a	29	0.15a	39	0.39	0.39	1.00
QB		0	50	250	500	500	144bcd	73	0.28abc	38	0.77	0.73	0.92
QC		0	50	250	500	500	148cd	38	0.59d	68	0.86	0.86	1.00
QL		0	50	150	300	300	187d	57	0.32abc	49	0.75	0.65	0.79
QS		0	50	250	500	500	131bcd	51	0.48bcd	63	0.82	0.76	0.88
RF		0	50	150	300	300	179cd	84	0.46bcd	80	0.58	0.58	1.00
WN		0	50	150	300	300	86ab	19	0.22ab	67	0.33	0.32	0.96
KD	Large	0	50	150	300	300	18a	4	0.30 cd	100	0.30	0.30^a^	—
LB		0	50	150	300	300	26ab	3	0.25abc	100	0.25	0.25^a^	—
NK		0	50	150	300	300	15a	10	0.11a	41	0.28	0.27	0.93
QB		0	50	250	500	500	27ab	13	0.26c	55	0.49	0.48	0.99
QC		0	50	250	500	500	30c	8	0.42d	87	0.50	0.48	0.74
QL		0	50	150	300	300	15a	4	0.27c	86	0.34	0.32	0.61
QS		0	50	250	500	500	23ab	5	0.43d	70	0.51	0.61^b^	—
RF		0	50	150	300	300	18a	7	0.25bc	100	0.25	0.25^a^	—
WN		0	50	150	300	300	19a	5	0.13ab	59	0.24	0.22	0.92

Five pre-germinated seeds of wheat (*Triticum aestivum L*., cv. Frame) were sown in each pot and thinned to two plants in the small pots and one in the large pots after one week, at the two-leaf growth stage. Soil moisture was maintained at approximately 65% of the water holding capacity throughout the experiment. A randomized block design was employed and pot positions were changed randomly twice a week within the block to minimize any effects due to spatial variations in growing conditions (e.g. light, temperature and humidity) within the glasshouse. During the course of the experiment, the temperature ranged from 22 to 24 °C and relative humidity ranged from 25 to 88%. Wheat plants were harvested 28 days after sowing, at the end of tillering (GS30) stage^39^. Since the root density obtained between treatments in each soil was similar (assessed visually), wheat roots were removed by washing from only one replicate of each K rate from both small pots and large pots. Roots were dried to constant weight, resulting in 4 replicate samples for each soil from which root density was calculated.

### Soil analyses

#### Extraction method details

Subsamples separated before planting were dried to constant weight at 40 °C and sieved to 2 mm before soil K testing. CaCl_2_-K was extracted using 0.01 mol L^−1^ CaCl_2_ at a soil to solution ratio of 1:10 for 2 h^40^; Colwell K was extracted using 0.5 mol L^−1^ NaHCO_3_ (pH 8.5) at a soil to solution ratio of 1:100 for 16 h^36, 41^; and exchangeable K (extracted using NH_4_OAc) was measured using 1 mol L^−1^ NH_4_OAc at a soil to solution ratio of 1:10 for 30 min^36^. Potassium in the eluents from the above tests was analyzed by an Inductively Coupled Plasma-Optical Emission Spectrometer (ICP-OES, PerkinElmer, Optima 7000DV) at λ of 766.490 nm.

#### DGT methods

A mixed Amberlite and ferrihydrite (MAF) gel was used as the binding gel to measure K and other cations. The MAF gel and diffusive gel were prepared as described previously^35, 42^. Standard DGT devices (DGT Research Ltd, Lancaster, UK) with an effective sampling area of 2.54 cm^2^ were used to load the gel assemblies (MAF gel, diffusive gel and filter paper). The absorbed cations on the resin gel were eluted and measured according to the methods described by Zhang *et al*.^43^. The concentration of K measured by the DGT method (C_DGT_) was calculated as described in the previous publications^35, 42, 43^.

### Plant relative dry matter and plant K analyses

The dry weight of aboveground plant parts was recorded after drying in an oven at 60 °C to constant weight. Maximum plant dry matter obtained for each soil was calculated by fitting a Mitscherlich response function in SigmaPlot 12.0 to describe the response in plant dry matter to applied K rates using Eq. (1):

1 $$D{M}_{max}=D{M}_{0}+a(1-\exp (-bx))$$

where *DM*_0_ is the wheat dry mass obtained in soils; *a* equals dry mass increase due to K application, *b* is the curvature coefficient resulting in (*DM*_0_ + *a*) equating to the maximum dry matter (*DM*_*max*_); relative dry matter (RDM) at each treatment was calculated as the ratio of dry matter yield to the *DM*_*max*_ obtained expressed as a percentage.

Plant tissue K concentration was analyzed using an acid digestion method^44, 45^. Plant samples (0.5 g, above ground part) were digested in 5 mL of nitric acid until the volume reduced to 1 mL, and then the solution was diluted to 20 mL for filtration. The filtered solution was then analyzed by an ICP-OES as outlined above. Overall plant K uptake was calculated by multiplying the dry mass by the tissue K concentration.

### Comparison of different soil testing methods

The accuracy of the soil testing methods for predicting wheat relative dry matter and K uptake across the different soils was compared using the coefficient of determination derived from fitting a Mitscherlich curve (Eq. 1) to the data. The critical value of each soil test method for indicating K deficiency was assessed as the value that delivered 90% of the maximum dry matter^46, 47^. When fitting the Mitscherlich curve, all curves were scaled to a relative basis by setting (*DM*_0_+a) to 100. The coefficient of determination was used to identify the most accurate soil testing method for predicting wheat dry matter response. For the DGT K method, any treatments where competing cations could have affected the measured value for K^43^ were excluded.

### Statistical analysis

Correlations between the values obtained from the different soil K test methods on control soils were assessed using the Spearman correlation method in the SigmaPlot 12.0 software. Analysis of variance (ANOVA) was performed using IBM SPASS Statistics 20 to assess whether the measured root density and dry mass were significantly different between soil types within each pot size.

## Results

### Correlations between soil tests

A significant correlation was obtained between the Colwell K and the NH_4_OAc K methods (R^2^ = 0.93, P < 0.01). However, no significant correlation (p ≤ 0.05) was found between the Colwell K or NH_4_OAc K methods and the DGT K method. The CaCl_2_ K method had moderate correlation with the Colwell K (R^2^ = 0.49, P < 0.01) and NH_4_OAc K methods (R^2^ = 0.51, P < 0.01), and correlated well with the DGT K method (R^2^ = 0.77, P < 0.01).

### Wheat responses to K

Contrasting root densities were obtained in small and large pots, as measured after wheat plants were harvested (Table 2). Significant differences in root densities were found between wheat roots in different soils in both small and large pots (P ≤ 0.05). Wheat grown in some control soils showed symptoms of K deficiency, i.e. yellowing tips and edges on leaves and stunted growth, reflecting low available K contents in those soils. Good wheat dry matter responses to K application were observed in most of the small pots (Table 2). Generally, wheat growth responses to K applications were larger in the small pots compared to that in the large pots, except for soils NK and WN. In the large pots containing soils KD, LB and RF, the response to K fertilizer application was negative, so *DM*_*max*_ was set at the dry mass in the control soil. In the large pots of soil QS, linear responses to K were observed, so *DM*_*max*_ was set as 120% of the dry mass for the highest K rate^48^. The predicted maximum dry matter was close to what obtained on each soil from the experiment (Table 2).

### Performance of soil tests to predict dry matter response to K fertilizer

**Figure 1:**
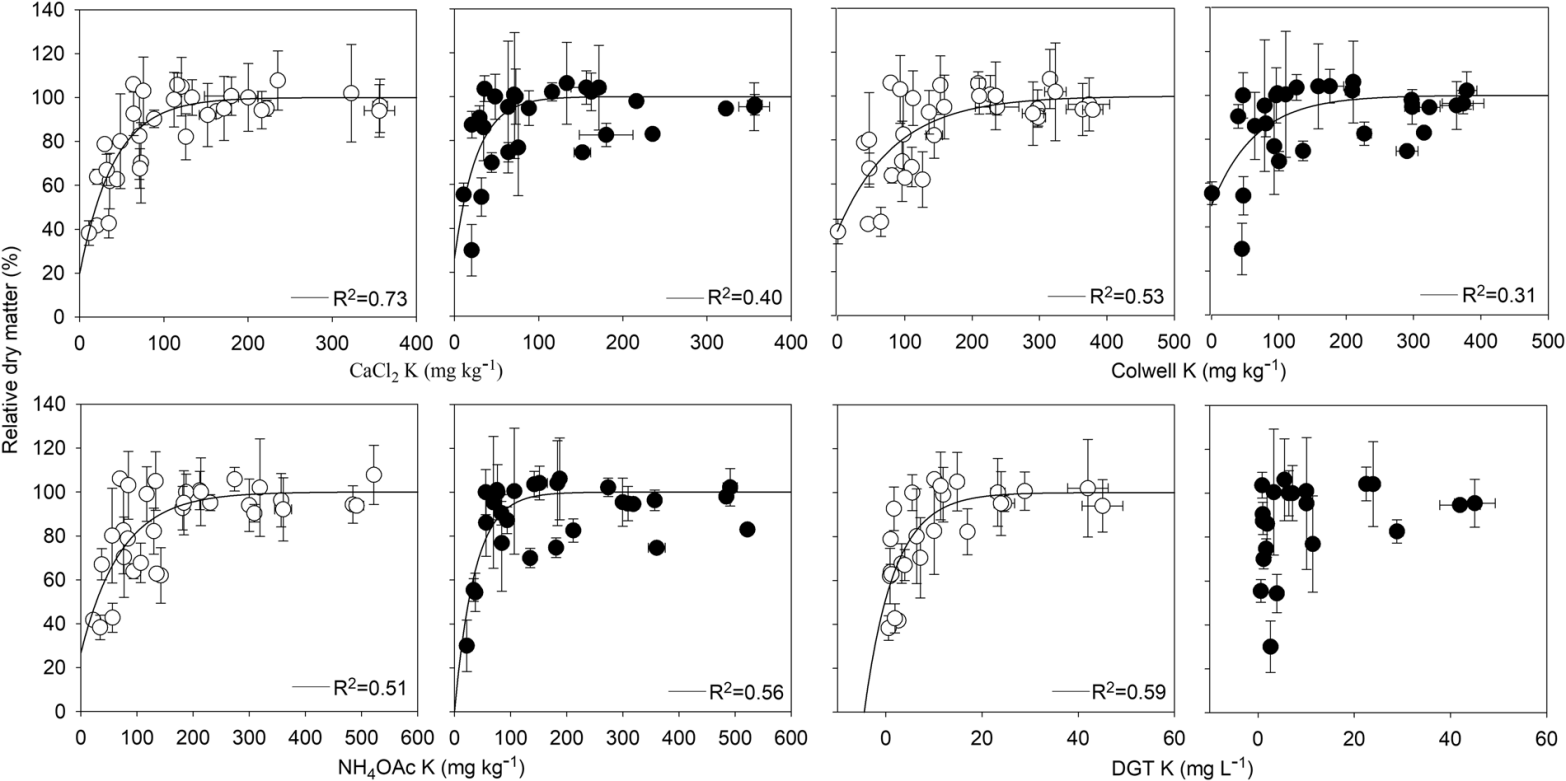
Relationship between extractable K with and the relative dry matter response of wheat to K fertilizer (open symbols represents the relative dry matter obtained from small pots and closed symbols represents the relative dry matter obtained from large pots).

The relationships between soil extractable K derived using the different methods and the relative dry matter response of wheat to K fertilizer in all soils are shown in Fig. 1. In the small pots, good relationships of wheat relative dry matter to extractable K values were obtained for the CaCl_2_ K method (R^2^ = 0.73), while all other tests relatively showed poorer relationships (R^2^ 0.51–0.59, Table 3). However in the larger pots, good relationships of wheat relative dry matter to measured soil K were obtained for the NH_4_OAc K method (R^2^ = 0.56, Fig. 1). CaCl_2_ K (R^2^ = 0.40) and Colwell K (R^2^ = 0.31) methods showed poor correlation relationships. The DGT K method performed poorly and no significant relationship (P ≤ 0.05) could be derived. The critical concentrations for different soil K test methods in all the soils at two root densities were shown in Table 3. Compared to the results obtained in all soils, the relationships between soil extractable K derived and the relative dry matter response of wheat to K fertilizer using different methods in control soils showed similar relationships. In the small pots, good relationships of wheat relative dry matter to extractable K values were obtained for the CaCl_2_ K method (R^2^ = 0.64), followed by the DGT K method (R^2^ = 0.57), while Colwell K and NH_4_OAc K methods showed no correlation relationships. In the larger pots, good relationships of wheat relative dry matter to measured soil K were obtained for the NH_4_OAc K method (R^2^ = 0.75), followed by the Colwell K (R^2^ = 0.70) method. CaCl_2_ K (R^2^ = 0.55) methods showed a relative poor correlation relationship and the DGT K method performed poorly and no significant relationship (P ≤ 0.05) could be derived.

**Table 3:** Critical values and coefficient of determinations for each test method for obtaining 90% of maximum dry matter at two root densities; “—” denotes no significant relationship was observed.

Soil test		CaCl_2_ K	Colwell K	NH_4_OAc K	DGT K
		mg kg^−1^			
High demand and root density	Critical value	83	151	142	10
	R^2^	0.73	0.53	0.51	0.59
Low demand and root density	Critical value	57	114	91	—
	R^2^	0.40	0.31	0.56	—

### Performance of soil tests to predict K uptake by plants

**Figure 2:**
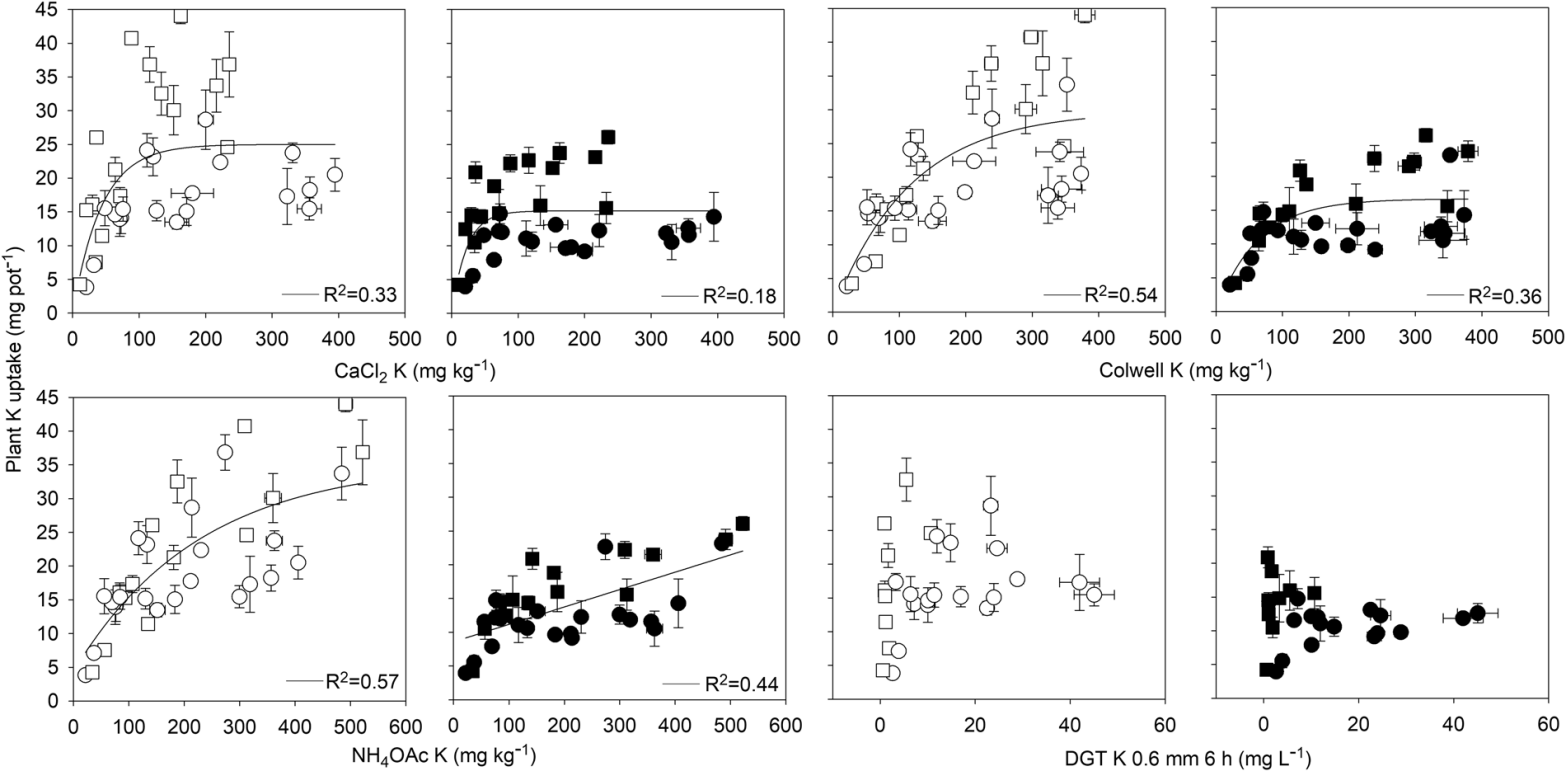
Relationship between extractable K and plant K uptake in wheat shoots grown in small (open symbols) or large (closed symbols) pots and receiving 4 rates of K fertilizer. Soils with CEC < 10 cmol kg^−1^ are represented by circles while square symbols represent soils with CEC > 10 cmol kg^−1^.

The amounts of K uptake (mg K pot^−1^) in the small pots were higher than that in the large pots due to more plants were grown in the small pots (Fig. 2). The coefficients of determination (R^2^) between extractable K and plant K uptake in wheat shoots were <0.44 for the large pots and <0.57 for the small pots (Fig. 2). NH_4_OAc K method was best able to predict plant K uptake (R^2^ = 0.57 in small pots; R^2^ = 0.44 in large pots), with a fairly similar level of accuracy found for the Colwell K method (R^2^ = 0.54 in small pots; R^2^ = 0.36 in large pots). The CaCl_2_ K method was less effective at predicting plant K uptake (R^2^ = 0.33 in small pots; R^2^ = 0.18 in large pots), while there was no correlation between the plant K uptake and the amounts of soil K extracted by the DGT method in each pot size. While using the control soils only, the best predictor was also the NH_4_OAc K method (R^2^ = 0.60 in small pots; R^2^ = 0.83 in large pots). A fairly similar level of accuracy was also found for the Colwell K method (R^2^ = 0.55 in small pots; R^2^ = 0.82 in large pots). The CaCl_2_ K method was less effective at predicting plant K uptake (R^2^ = 0.44 in small pots; R^2^ = 0.54 in large pots), while there was no correlation between the plant K uptake and the amounts of soil K extracted by the DGT method in each pot size.

### Relationship between K concentration in shoot and relative dry matter

**Figure 3:**
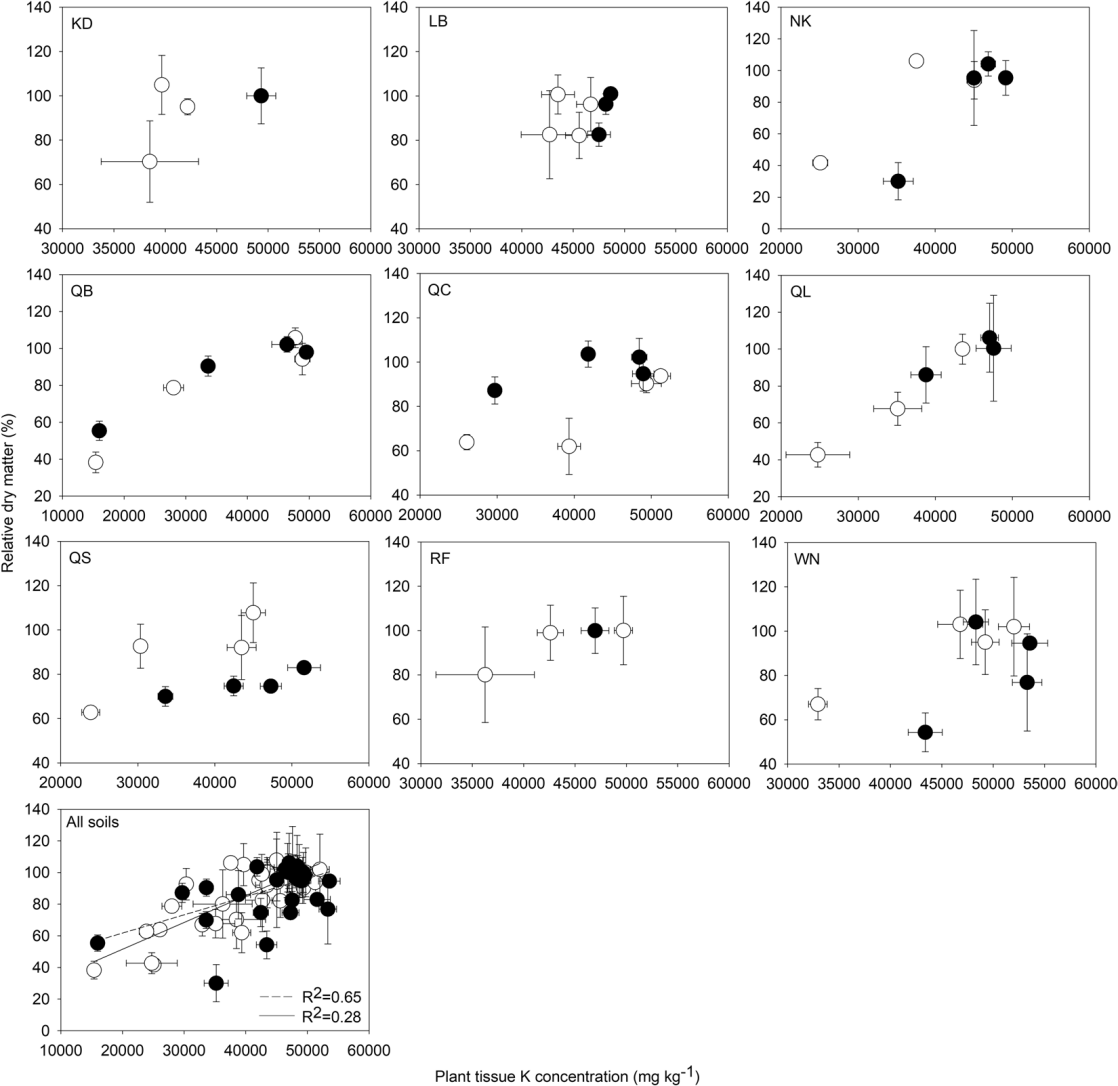
Relationships between concentrations of K in shoots and relative dry matter for wheat grown in 9 contrasting soil types (open symbol represent the values obtained from small pots and closed symbols represent the values obtained from large pots). The final figure is a combined analysis across all 9 soil types, with the dashed and continuous lines representing the relationship curve for small pots and large pots, respectively. R^2^ in the large pots rises from 0.28 to R^2^ = 0.63 when soils NK and WN were excluded from the results.

Generally, higher relative dry matter was observed in plants with higher K concentrations in each soil (Fig. 3). A moderate correlation between wheat relative dry matter and plant tissue K concentrations was observed in the small pots (R^2^ = 0.65), with 42600 mg K kg^−1^ required to obtain 90% relative dry matter. Soils NK and WN seemed to behave differently under the different root densities and were the only two soils where responses to K were larger in the large pot-low root density conditions. The relationship between dry matter and tissue K concentration in large pots was improved when soils NK and WN were excluded from the results, rising from an R^2^ = 0.28 to R^2^ = 0.63. The overall critical tissue concentration to obtain 90% relative dry matter was 42800 mg kg^−1^ for the plants in the large pots, which was very similar to the critical value obtained in the small pots.

## Discussion

### Correlations between soil tests

Based on the correlations between soil K testing methods, they can be categorized into 3 groups: 1) CaCl_2_ K method, 2) exchangeable K methods (Colwell K and NH_4_OAc K) and 3) DGT K method. A high correlation between the Colwell K (exchangeable K) and NH_4_OAc K (exchangeable K) methods (slope of NH_4_OAc K/Colwell K = 1.11, R^2^ = 0.93, P < 0.01) suggests that these two methods measure a similar K pool, commonly referred to as exchangeable K. While both extractants in effect measure both soil solution K and readily desorbable K on exchange sites, the former is usually an extremely small fraction of the quantity measured– except where recent fertilizer or manure applications have been made^49^. The bulk of K measured in these tests therefore refers to the pool of K that is in a form immediately available for movement into the soil solution in response to depletion by plant root uptake^50^. In soils that have limited mineral K sources, exchangeable K is therefore effectively an index of the capacity of the soil to supply bioavailable K over an extended period (a quantity factor). The close correlation between the Colwell K and NH_4_OAc K methods extraction methods is consistent with findings from the review by McLean and Watson^4^. Although the soil to solution ratio higher and extracting period is shorter than that for the Colwell K method, the slight increase in extraction efficiency with the NH_4_OAc K method is mainly due to the similarity in hydrated radius of the NH_4_^+^ and K^+^ and a higher extraction concentration, allowing more complete displacement of K from the jagged edges and interlayer positions in tetrahedral sheets of clay minerals.

The lower concentration and larger size of Ca^2+^ relative to Na^+^ and NH_4_^+^ mean that the CaCl_2_ method is much less effective at displacing K from tightly held exchange positions on the solid phase, meaning the measurement is more closely related to soil solution K (a measure of the intensity of K supply) than to exchangeable K. The lesser but still significant (R^2^ = 0.49–0.51) correlation between the CaCl_2_ K method and the Colwell K and NH_4_OAc K methods may be at least partly due to the effect of recent fertilizer applications disproportionately increasing the fraction of Colwell K and NH_4_OAc K extracts that consists of soil solution K. Soils with high buffer power (typically high cation exchange capacity) hold increasing proportions of K tightly bound on soil solid phase, even with fresh fertilizer additions. While this K can be measured by the NH_4_OAc or Colwell K methods, it cannot be measured by solution K methods (e.g. CaCl_2_ K method). So the differences between the quantity and intensity extraction methods are likely to increase as CEC and soil buffer power increase.

The moderate correlations between the DGT K method and the CaCl_2_ K method (R^2^ = 0.77, P < 0.05) are consistent with the fact that both are defacto measures of soil solution K (a surrogate K intensity factor), in agreement with Zhang *et al*.’s result^43^. However the DGT K method adds a diffusion factor purported to better relate to the rate of diffusive supply to a DGT surface upon dilution of the solution K phase and K resupply from the soil solid phase. Furthermore, the extracting force of K from soils with high CEC by DGT K method is weak, as lower C_DGT_ values were obtained on soils with higher CEC values which were amended with more K before wheat growing (last component in Fig. 2). This may explain the lack of correlation between the DGT K results and those of Colwell K and NH_4_OAc K methods (in contrast to that for CaCl_2_ K method).

### Performance of soil tests to predict relative dry matter and K uptake

The results obtained by using all soils and control soils were comparable because the relative dry matter and K uptake response to soil testing values using different extracting methods at two root densities showed similar accuracy. The wheat roots were constricted in the pots in the glasshouse trial and a net-like root mat was observed at the bottom of all pots, but particularly for the small pots. Due to the limited soil volume and increased plant number in the small pots, there is the likelihood that the available K in the small pots was exhausted, and that plant growth was restricted before the biomass was measured compared to the plants in the large pots. It was reported that root competition could cause a reduction in the availability of a soil resource^51^. Root volume confinement could also result in an increased abscisic acid concentration within root system and decreased dry weight in biomass^52, 53^. The root density of winter wheat in a field experiment (soil depth ≤ 20 cm) was reported to be around 0.15 g cm^−3^ by Yao *et al*.^54^, which was close to the root density obtained from the large pots in this study. Therefore, we assumed that the root density in the large pot was more representative of the field conditions than that in the small pots.

The present study showed good correlations between wheat relative dry matter and K extracted by the NH_4_OAc K method at lower root densities. The critical value (90% relative dry matter) for the NH_4_OAc K method was 91 mg kg^−1^ at low root density (large pots) in all soils. While there are few reported studies developing critical soil test K values using the NH_4_OAc K method in the literature, Brennan and Bell reported a critical Colwell K critical value of 40–64 mg kg^−1^ for grain yield of wheat in field condition, with the range related to contrasting soil types^55^. This value was much lower than the 114 mg K kg^−1^ value we derived in this study, with the difference attributed to differences in plant growth stage, root density, water content and access to greater soil volumes (e.g. subsoil K) by roots in field conditions. Regardless, our data suggest the NH_4_OAc K methods were more accurate than other methods for predicting wheat response to K applications. The difference in critical values of soil K test is influenced by the ratio of other cations and their plant uptake. Also, the rate of K supply from less available soil K pools may not be sufficient to meet crop demand where rooting patterns or slow diffusion limits the supply to the crop especially in vertisols. This suggest the estimation of reliable critical soil K test values still needs further investigation for grain cropping soils of Australia.

For prediction of K uptake, Colwell K and NH_4_OAc K methods seemed to be more accurate than other methods in control soils at low root densities, with this difference maintained when freshly fertilized soils were included in the assessment (R^2^ = 0.36 for the Colwell K and R^2^ = 0.44 for the NH_4_OAc K method) (Fig. 2). In the small pots, two plants with larger biomass generated a high K demand, and the high density plant roots were able to take up nearly all the available K from soils. Consequently, diffusion and/or soil supply rate contributes very little to K uptake by plants in the small pots, but the total available K amounts in soils represent plant acquisition at high demand and root density. Therefore, the NH_4_OAc K method should best predict K uptake by plants at high root density, as the NH_4_OAc K method is considered the quantity factor for soil available K measuring. The accuracy of predicting K uptake also decreased as K demand decreased in the large pots. On the contrary, plants took up K easily in a large pot compared to that in a small pot. Because there was no high K demand produced by aggressive plant biomass, plants took up K in a limited space around the roots, representing the dynamics associated with nutrient supply and plant acquisition. In the large pots, plant roots continued to be able to explore “new” space in the pots with relative sufficient K^+^ presented in the soil solution, where diffusive supply factors also become more notable as K^+^ diffused from “new” soil to exploited soil. The performance of DGT for predicting K uptake was poor at high root density, but the performance was also unexpectedly poor at low root density as well. Compared to other methods, high K uptakes by plants from soils with high CEC values (soils with CEC >10 cmol kg^−1^ as shown in square symbols in Fig. 2) was observed, but the measured DGT K values were low. That is to say, there was a proportion of available K in soils that can’t be measured by the DGT K method, but had been used by plants. Therefore, we can concluded that the extraction force of the DGT device for K is too weak on the soils with high CEC values and DGT K method may not be a suitable soil test method to assess the plant available K in certain soil types.

### Effects of root density on critical values for soil tests

The performance of the soil tests to predict response to K fertilizer was affected by the different root densities induced by varying pot size. The critical value increased with increased root density (Table 3). The reason is presumably that higher root density creates a stronger demand on soil K pools thereby requiring a greater amount of available K to satisfy this demand. This indicates that calibrating soil tests with crop fertilizer requirements for K is difficult under glasshouse conditions as root densities are often higher than in the field, so critical values determined under glasshouse conditions are unlikely to accurately predict responses in the field. These inaccuracies will increase as pot size decreases.

We hypothesized that at high root density (small pots) the capacity measures (Colwell K and NH_4_OAc K methods) would correlate better with plant response due to significant K depletion, while at low rooting density it was more likely that intensity measures of soil K (CaCl_2_ and DGT) might correlate well with plant response to K fertilizer. However, the intensity measures of soil K unexpectedly showed worse correlations with plant response (both dry matter and K uptake) to K fertilizer compared to the capacity measures at low root density. There was also the likelihood that K demands by plants in the small pots were larger than that soils could supply due to limited soil volume/mass employed, thereby being the reason that more response of wheat relative dry matter to K application was observed.

### Plant K concentration relationship with relative dry matter

Although plant K concentration in tissue can vary with time and in different parts of the same plant^56, 57^, K concentration of the whole plant (dry mass basis) stays at a similar level at the same stage of plant growth^58^. Moderate correlations between wheat relative dry matter and tissue K concentration (R^2^ = 0.65 at high root density; R^2^ = 0.63 at low root density when soils NK and WN were excluded) were observed across the soils (Fig. 3). A consistent correlation relationship of wheat relative dry matter and tissue K concentration at different root densities suggests that plant tissue K concentration could be used to infer wheat growth constraints in terms of K limitations for diagnose purposes. The correlation relationship deteriorated when values from soils NK and WN were included in the analysis, resulting in a lowered coefficient of determination in the low root density situation. The failure to achieve a strong growth response to K in soils NK and WN was probably due to some unpredicted factors during the growth period in the large pots (e.g. poor aeration, or water accumulation at the bottom of the pots).

## Conclusions

This study investigated four soil testing methods to predict wheat response to K application in 9 agricultural soils in a glasshouse trial under two root densities. The predictive accuracy of soil K testing methods varied with root density. Comparing the plant growing conditions to that in the field, the low root density of plant roots in large pots was a more realistic approximation of field conditions. In these larger pots the NH_4_OAc K method had the highest accuracy for predicting wheat relative dry matter and plant K uptake in response to K application across all soils. The critical value for 90% wheat relative dry matter at the end of tillering (GS30) stage for the NH_4_OAc K method was 91 mg kg^−1^. Although the DGT technique measures elements in soil solution and parts from the soil solid phase, it failed to accurately predict wheat relative dry matter or plant K uptake responses to K applications, presumably because the extraction force of K from soils by the DGT was too weak compared to that by plant roots. Further study is needed to validate the performance of these soil K test methods in field conditions.
